# Prescription Appropriateness of Drugs for Peptic Ulcer and Gastro-Esophageal Reflux Disease: Baseline Assessment in the LAPTOP-PPI Cluster Randomized Trial

**DOI:** 10.3389/fphar.2022.803809

**Published:** 2022-03-28

**Authors:** Ilaria Ardoino, Manuela Casula, Giulia Molari, Sara Mucherino, Valentina Orlando, Enrica Menditto, Carlotta Franchi

**Affiliations:** ^1^ Laboratory of Pharmacoepidemiology and Human Nutrition, Department of Health Policy, Istituto di Ricerche Farmacologiche Mario Negri IRCCS, Milan, Italy; ^2^ IRCCS MultiMedica, Sesto S. Giovanni, Milan, Italy; ^3^ Epidemiology and Preventive Pharmacology Service (SEFAP), Department of Pharmacological and Biomolecular Sciences, University of Milan, Milan, Italy; ^4^ CIRFF, Center of Pharmacoeconomics and Drug Utilization Research, Department of Pharmacy, University of Naples Federico II, Naples, Italy

**Keywords:** drugs for peptic ulcer and gastroesophageal reflux disease, proton pump inhibitors, gastroesophageal reflux disease, appropriateness of drug use, algorithm, administrative database, older people, educational intervention

## Abstract

**Background:** Drugs for peptic ulcer and gastro-esophageal reflux disease (GERD) are among the most widely prescribed, frequently without appropriate indications. This represents an important issue, as it leads to risk of adverse events for patients and unnecessary costs for National Health Service.

**Aim:** To assess the prescription appropriateness of drugs for GERD, in the frame of the “Evaluation of the effectiveness of a Low-cost informative intervention to improve the Appropriate PrescripTiOn of Proton PumP Inhibitors in older people in primary care: a cluster-randomized controlled study” (LAPTOP-PPI) (Clinicaltrial.gov: NCT04637750).

**Methods:** The appropriateness of drug prescription was assessed on data collected in administrative databases, by integrating information on concomitant medications, outpatient medical and laboratory procedures and hospital discharge diagnoses, according to the reimbursement criteria provided by the Italian Medicine Agency. We analyzed data of community-dwelling people aged 65 years and over, living in the areas of Bergamo (Northern Italy) and Caserta (Southern Italy), from July 1 to 31 December 2019.

**Results:** Among 380,218 patients, 175,342 (46.1%) received at least one prescription of drugs for GERD. All in all, we found that only 41.2% of patients received appropriate prescriptions.

**Conclusion:** Given the potential risk of adverse drug reactions, especially in older people, educational interventions should be prompted for physicians, in order to improve the quality of prescription of drugs for GERD and, in turn, avoid unfavorable health outcomes and unnecessary costs.

## Introduction

Gastro-esophageal reflux disease (GERD) is common worldwide, accounting for a global prevalence of 14%, and Italy is among the countries with the highest percentage (Nirwan et al., 2020; GBD 2017 Gastro-oesophageal Reflux Disease Collaborators, 2020). Drugs for GERD include antacids, H2 receptor antagonists (Anti-H2), Proton Pump Inhibitors (PPIs) and prostaglandins (Gyawali and Fass, 2018). PPIs are the most common prescribed medications to treat GERD, to prevent antiplatelet or non-steroidal anti-inflammatory (NSAID) drug-induced ulcer, to eradicate *Helicobacter pylori* (HP), and to treat Zollinger-Ellison syndrome (Scarpignato et al., 2016; Savarino et al., 2018a). In 2019, in Italy, the overall use of drugs for GERD was 80.5 Defined Daily Dose (DDD)/1000 inhabitants/die, with an increase of 1.5% compared to 2018 (AIFA- Italian Medicine Agency, 2019). In particular, PPIs, with 72.6 DDD/1000 inhabitants/die, accounted for 90% of consumption of this drug class, and increased of 2.5% compared to the previous year (AIFA- Italian Medicine Agency, 2019). Although drugs for GERD, and PPIs in particular, are characterized by an undisputed efficacy and tolerability, evidence shows that, in different settings, they are often prescribed without proper indication (Wallerstedt et al., 2017; Jaynes and Kumar, 2018; Nguyen and Tamaz, 2018; Voukelatou et al., 2019; Lenoir et al., 2019; Franchi et al., 2020), exposing patients to an increased risk of adverse drug reactions (ADRs), such as hypomagnesaemia, *Clostridium difficile* infection, pneumonia, chronic kidney disease, and fractures (Jaynes and Kumar, 2018). Inappropriate prescriptions represent a matter of concern especially in older patients, who are often affected by multiple chronic diseases, exposed to polypharmacy and thus at further increased risk of potential drug-drug interactions and ADRs (Franchi et al., 2016a; Franchi et al., 2019a; Antoniazzi et al., 2019). Despite this, the use of drugs for GERD increases with age, reaching the prevalence of 60% in the 75 years or older people (AIFA- Italian Medicine Agency, 2019). Furthermore, the inappropriate use of these medications results in increment of both direct and indirect healthcare costs. This critical framework prompted the National Health Services (NHS) worldwide to suggest criteria aimed at promoting the appropriateness of drug use and the restraint of healthcare costs (AIFA- Italian Medicine Agency, 2019). Moreover, the promotion of appropriate drug prescription become the goal of several educational clinical trials in different settings (Franchi et al., 2016b; Davey et al., 2017; Savarino et al., 2018b; Nguyen-Soenen et al., 2020; Franchi et al., 2021).

With this background, we designed a cluster-randomized controlled trial to promote the appropriate use of drugs for GERD in primary care of older population. Below, we report the results on appropriateness of use of drugs for GERD during the trial baseline period by using data obtained from administrative database of two local health units (LHUs) of Northern and Southern Italy.

## Methods

### LAPTOP-PPI Study Design

We designed a cluster randomized controlled trial “Evaluation of the effectiveness of a Low-cost informative intervention to improve the Appropriate PrescripTiOn of Proton PumP Inhibitors in older people in primary care: a cluster-randomized controlled study (LAPTOP-PPI)” aimed at evaluating the effectiveness of a low-cost informative intervention, addressed to General Practitioners (GPs) in the LHUs of Bergamo (northern Italy) and Caserta (southern Italy). The final goal was to reduce the inappropriate over-prescription of drugs for GERD among community-dwelling older people. LAPTOP-PPI has been structured in four phases: 1) baseline period, in which the appropriateness of prescriptions of drugs for GERD in patients aged 65 years and older was assessed using data collected in administrative databases; 2) randomization, in which every GP still working in the LHUs was randomized to intervention or control arm according to the results of the baseline assessment; 3) intervention period, in which every GP randomized to the intervention was informed with summary statistics on his/her prescribing habits (e.g. number of patients prescribed with drugs for GERD and the proportion of patients appropriately prescribed referring to the baseline period, compared with the data pertaining the respective LHU). In addition, we provided GPs with an educational summary on how to safely prescribe or deprescribe drugs for GERD, according to the criteria proposed by Italian Medicine Agency (AIFA). Four months after the randomization, the GPs in the intervention arm also received posters to hang in their ambulatory and leaflets to distribute to their patients, in order to sensitize and empower them to properly use GERD medications. GPs randomized to the control arm carried on with their current clinical practice. 4) post-intervention period, in which the effectiveness of the intervention will be reassessed using the new data collected in administrative databases at 6, 12 and 18 months after randomization, in order to verify the reduction of over-prescriptions of drugs for GERD.

### The NHS Criteria for Prescription Appropriateness: The NOTE AIFA

In Italy, the NHS guarantees minimal levels of assistance to all citizens and full or partial reimbursement for specific drugs. In 1993, AIFA introduced a regulatory tool for the control of pharmaceutical expenditure for specific drug categories, based on evidence from guideline, known as NOTE AIFA. (AIFA- Italian Medicine Agency, 2019); Savarino et al., 2018b) The NOTE AIFA define the therapeutic indications and/or the categories of patients for whom the drugs have a proven efficacy and an optimal risk-benefit ratio, thus worthy to be fully or partially reimbursed by NHS. Reimbursement criteria for drugs for peptic ulcer and GERD (PPIs, Anti-H2, and prostaglandins) are summarized in NOTA 1 and 48 (Table 1).

**TABLE 1:** Rules of the Italian Drug Agency for the reimbursement of proton pump inhibitors (PPI) and other drugs for GERD.

—
NOTA 1—Reimbursement from the National Health Service of prescriptions of PPIs and Misoprostol is limited to
The prevention of serious complications of the upper gastrointestinal tract in patients on chronic treatment with NSAIDS or on antiplatelet therapy with low doses of ASA for cerebro or cardio-vascular disease prevention, provided there is one of the following conditions of risk
• history of past digestive hemorrhages or peptic ulcer
• concomitant therapy with anticoagulants or corticosteroids
• advanced age
NOTA 48—Reimbursement of PPIs and H2-receptor antagonist prescription by the National Health Service is limited to the following periods and conditions
Duration of treatment 4 weeks (occasionally 6 weeks)
• Duodenal or gastric ulcer, in association with drugs eradicating the infection
• GERD with or without esophagitis (first episode)
Extended duration of treatment, to reevaluate after 1 year
• Zollinger-Ellison syndrome
• relapsing duodenal or gastric ulcer
• GERD with or without esophagitis (relapsing)

PPIs: Proton pump inhibitors; GERD: Gastro-esophageal reflux disease; ASA: acetylsalicylic acid; NSAIDS: Non-steroidal anti-inflammatory drugs.

### Data Collection

We analyzed data obtained from the administrative databases of Bergamo and Caserta LHUs.

The administrative databases were routinely updated for administrative and reimbursement purposes, and were structured as follows: 1) sociodemographic and administrative data of every patient assisted by the Italian NHS; 2) data on drugs prescribed (and identifiable according to the Anatomical Therapeutic Chemical (ATC) classification system) by the GPs or by hospital physicians and partially or entirely reimbursed by the NHS and dispensed through the community pharmacies, 3) data on Hospital Discharge Records reporting the main diagnosis, and up to other five comorbidities (codified according to International Classification of Diseases, Ninth Revision, Clinical Modification (ICD-9- CM) classification), other than diagnostic and therapeutic procedures implemented during the episodes of hospitalization provided in public and private hospitals; 4) data on outpatient services such as ambulatory specialists’ visits, diagnostic procedures and laboratory tests that were provided by health providers accredited with the NHS and 5) national exemption codes that ascertain acute and chronic conditions for which patients were assisted entirely free of charge [https://www.salute.gov.it/portale/esenzioni/dettaglioContenutiEsenzioni.jsp?lingua=italiano&id=1017&area=esenzioni&menu=vuoto].

Record linkage between different sources was allowed by a national unique identification code assigned to each beneficiary of the NHS. To preserve privacy, identification codes were automatically converted into anonymized codes, and the conversion table was stored by the regional authorities that manage the data. The structure of these databases has been described in more details elsewhere (Franchi et al., 2021).

The project was approved by the ethical committees of Campania Nord and Bergamo LHUs and all data were managed according to the current Italian law on privacy.

### Study Population

All community-dwelling patients aged 65 years or more who lived in the areas of Bergamo and Caserta and who received at least one drug prescription in the period from 01/07/2019 to 31/12/2019 were scrutinized, regardless of whether or not they received drugs for peptic ulcer and GERD (ATC: A02B*). The number of days covered by each dispensed drug was calculated by dividing the total amount of active substance in each package by the defined daily dose (DDD).

### Algorithm to Assess the Appropriateness of Prescriptions of Drugs for Peptic Ulcer and GERD

In order to identify patients prescribed according to NOTA AIFA 1 and 48, we developed the following algorithm, which is able to capture information from administrative databases (Supplementary Figure S1).

NOTE AIFA 1 and 48 consider only PPIs (ATC: A02BC*), Anti-H2 (ATC: A02BA*), or prostaglandins (ATC: A02BB*), thereby we excluded from our assessment patients prescribed only with other drugs for GERD (A02BX*) or with the combinations for the eradication of *Helicobacter* Pylori (A02BD*).

Firstly, patients were divided into occasional, short-term, and chronic users. Occasional users were those patients who received only one prescription of drugs for GERD for no more than 28 days of treatment in the baseline period (01/07/2019-31/12/2019). Short-term users were those who received 29–60 days of treatment, while chronic users were those with at least 60 days of treatment. We referred to the date of first prescription with a drug for GERD as index date.

Occasional users were considered as appropriately prescribed regardless of compliance with the NOTE AIFA, as they may have actually been prescribed on demand to relieve pain, when specific symptoms occur. Appropriateness of prescription was assessed for all remaining patients according to NOTA one criteria, and then according to NOTA 48 as follows:

### NOTA 1

The conditions in NOTA one were verified for both short-term and chronic users.

We firstly checked the following requirement

A. In the period starting from 180 days before the index date up to 31/12/2019, being prescribed with both low-dose ASA (ATC: B01AC06 N02BA01), and nonsteroidal anti-inflammatory drugs (NSAIDs - ATC: M01*) for at least 90 days each (also not consecutive).

In case condition A was not satisfied, we checked for

B. In the same period of assessment, being prescribed with or ASA or NSAIDs for at least 90 days.

In addition to condition B, at least one of the following conditions had to occur during the same period of assessment:

1. Being prescribed at least 90 days with corticosteroids (ATC: H02* or R01AD*) or other anticoagulants (ATC: B01*, excluded ASA and Vitamin K antagonists);
2. Having received two or more packages of Vitamin K antagonists;
3. Being aged 75 years or more (Franchi et al., 2020);
  or within 24 months before the index date:
4. Being discharged from hospital with a diagnosis of *Helicobacter* Pylori (HP) infection or of peptic ulcer or hemorrhages of esophagus, stomach, or duodenum (ICD-9-CM codes in Supplementary Table S1);
5. Undergoing to diagnostic tests to ascertain the presence of peptic ulcer, GERD or HP infection (ICD-9-CM codes in Supplementary Table S1).

For patients with index date from 01/10/2019 to 31/12/2019, the prescription with drugs mentioned above (e.g. ASA, NSAID etc.) except those for GERD was assessed up to 31/03/2019, in order to avoid bias due to different time exposures.

A diagnosis of specific chronic conditions that may entail a long-term treatment with NSAIDs was considered as an alternative of having prescriptions with NSAIDs both in condition A and B (Supplementary Table S1).

### NOTA 48

Patients chronically prescribed and not adherent to NOTA 1 were generally considered as not appropriately prescribed, as NOTA 48 recommends short-term treatment for the cure of peptic ulcer or GERD. Indeed, ICD-9 codes do not distinguish between first episode and acute GERD from chronic disease, for which longer treatment could be considered. However, we checked for the occurrence of Zollinger-Ellison syndrome among all patients according to the following conditions in the 24 months preceding the index date:

C. Hospitalization for Gastrinoma or Zollinger-Ellison syndrome (Supplementary Table S1),
D. Undergoing to surgical intervention for incision or removal of the stomach (Supplementary Table S1).

For short-term GERD drug users only, we checked the fulfillment of at least one of the followings:

E. Previous condition 4 or 5;
F. Being prescribed with at least two antibiotics among: amoxicillin (ATC: J01CA04, J01CR02), metronidazole (J01XD01), clarithromycin (J01FA09), levofloxacin (J01MA12) or tetracycline (J01AA07), being generally required for the eradication of HP infections, within a range of 14 days before or after the date of any PPI prescription (Kim and Chung, 2020).

Patients who have been prescribed with combinations for eradication of HP (ATC: A02BD*), in combination with other drugs for GERD, were considered appropriately prescribed, regardless of the duration of treatment (Kim and Chung, 2020).

We considered both over- and under-prescription, thus we also identified patients who would have had an indication of treatment with drugs for GERD, but were not actually prescribed. For this purpose, we used the same algorithm, but we did not consider the concomitant use of antimicrobials for the eradication of HP, which may have too many indications of treatment.

## Results

Overall, 380,218 subjects aged 65 years or more in the LHU of Bergamo and Caserta received at least one drug prescription and were eligible for the present analyses: 219,622 in Bergamo and 160,596 in Caserta. Of these, 175,342 (46.1%) subjects, specifically 80,684 (36.7%) in Bergamo and 94,658 (58.9%) in Caserta, received at least one drug for peptic ulcer and GERD during the baseline period. Among the drugs for GERD, the most prescribed class is that of PPIs both alone and in combination (N = 171,106 – 97.6%). In particular, the median number of DDD prescribed for PPIs in Bergamo was 98 (Inter Quartile Range (IQR): 56–168), and in Caserta was 112 (IQR: 56–168). Patients’ characteristics were summarized in Table 2.

**TABLE 2:** Patient characteristics according to geographical areas of Northern and Southern Italy.

	Bergamo		Caserta		Total	
Variable	**N**	**%**	**N**	**%**	**N**	**%**
Total	80,684	—	94,658	—	175,342	—
Sex						
Male	34,724	43.0	40,216	42.5	76.4 (7.5)	42.7
Female	45,960	57.0	54,442	57.5	100,402	57.3
Age						
Mean (St.dev.)	77.2 (7.5)	—	75.8 (7.3)	—	—	—
65–74 years	32,471	40.2	46,214	48.8	78,685	44.9
75–84 years	33,208	41.2	35,139	37.1	68,347	39.0
≥85 years	15,005	18.6	13,305	14.1	28,310	16.1
Frequency of PPI use						
Only HP Combination	3	0.0	1	0.0	4	0.0
Only Other Drugs for GERD	1,565	1.9	1,795	1.9	3,360	1.9
Occasional Users	9,273	11.5	11,799	12.5	21,072	12.1
Short Term Users	13,030	16.1	12,953	13.7	25,983	14.8
Chronic Users	56,813	70.5	68,110	71.9	124,923	71.2

HP: helicobacter pylori, GERD: Gastro-esophageal reflux disease.

Overall, 3364 (1.9%) patients were prescribed only with combinations for the eradication of HP or with other drugs (such as alginates) not included in the indication of NOTA 1 and 48. Finally, our analysis was performed on 171,978 patients.

Table 3 shows detailed results on the appropriateness of GERD drug consumption for the two LHUs (overall and separately). Out of 171,978 patients, 70,785 (41.2%) were appropriately prescribed, of which 21,072 (29.8%) were occasional users and therefore immediately considered as appropriately prescribed. Finally, 150,906 (86.1%) were assessable for NOTA 1 and 48. Among short-term and chronic users, 49,713 out of 150,906 (32.9%) patients can be considered appropriately prescribed. In both areas, almost all appropriate prescriptions were administered according to the criteria of NOTA 1, to prevent gastrointestinal bleeding in high-risk patients, chronically prescribed with low doses ASA or NSAIDs, particularly due to the age of 75 years or more.

**TABLE 3:** Appropriateness of Proton Pump Inhibitors (PPIs) prescription according to geographical areas.

—	Bergamo		Caserta		Total	
—	(**N = 79,116**)	—	**(N = 92,862)**	—	**(N = 171,978)**	—
Appropriate	**36,408 (46.0%)**	—	**34,377 (37.0%)**	—	**70,785 (41.2%)**	—
Occasional users	**9273 (25.5%)**	—	**11,799 (34.3)**	—	**21,072 (29.8%)**	—
ASA or NSAIDs users	33,504	—	55,269	—	88,773	—
Of which assessable for NOTA1 (≥90 days)	24,469		32,472		56,941	
Appropriate for NOTA 1 - ASA or NSAIDS users (≥90 days)	**22,107 (60.7%)**	—	**21,786 (63.4%)**	—	**43,893 (62.0%)**	—
(A) ASA and FANS	—	1126	—	3106	—	4232
(B1) Corticosteroids	—	1264	—	949	—	2213
(B1) Other Anticoagulants	—	4015	—	4328	—	8343
(B2) Vit. K Antagonist	—	1112	—	257	—	1369
(B3) Old (≥75 years)	—	15,920	—	17,462	—	33,382
(B4/5) GERD	—	9014	—	1068	—	10,082
HP (coprescription with A02BD)	—	7	—	11	—	18
	Appropriate for Nota 48—Curative intent	**6517 (17.9%)**	—	**1029 (3.0%)**	—	**7546 (10.7%)**	—
(C/D)Zollinger-Ellison syndrome		—	693	—	565	—	1258
	Assessable for NOTA 48 (≤60 days)	13,030	—	12,953	—	25,983	—
(E) GERD	—	5836	—	410	—	6246
HP (coprescription with A02BD*)	—	11	—	10	—	21
(F) HP °	—	124	—	77	—	201
Not Appropriate	**42708 (54.0%)**	—	**58485 (63.0%)**	—	**101,193 (58.8%)**	—

ASA: acetylsalicylic acid, NSAIDs: nonsteroidal anti-inflammatory drugs, GERD: Gastro-esophageal reflux disease, HP: helicobacter pylori

When co-prescribed with double antibiotic therapy.

Capital letter in parenthesis referred to the condition explained in the section “Algorithm to assess the appropriateness of prescriptions of drugs for peptic ulcer and GERD” in the Methods.

The sum of criteria concerning “occasional users”, “Nota 1” and ‘Nota 48’—in II, IV, VI, column -, and the sum of the conditions in NOTA 1 and NOTA 48—in III, V, VII, column - exceeds 100% because a patient may be appropriately prescribed according to criteria both in NOTA 1 and 48.

The bold values represent the total number of patients presenting the condition reported in the header of the row on the left.

All in all, only 7546 (10.7%) patients had a proper indication for NOTA 48, with large differences among the LHUs. In particular, among short-term users in Caserta only few patients (N = 487 – 3.8%) had a confirmed or suspected diagnosis of GERD or of other predisposing conditions that provide an indication for this treatment. Also, the proportion of patients in Caserta appropriately prescribed according to NOTA one due to a diagnosis of GERD were very low.

Globally, 204,876 out of 380,218 (53.9%) subjects did not receive prescriptions for drugs for peptic ulcer or GERD: 138,938 (67.8%) in Bergamo and 65,938 (32.2%) in Caserta. Among these, we found 29,510 (14.4%) patients with a possible indication to receive drugs for GERD, 25,105 (85.1%) in Bergamo and 4,405 (14.9%) in Caserta.

## Discussion

We provided the results of the baseline period of the LAPTOP-PPI trial, aimed at assessing the appropriateness of prescription of drugs for GERD in community-dwelling older people in Italy, using a novel algorithm that acquires information from administrative databases. The appropriateness criteria were established on the indications provided by the reimbursement criteria of the Italian Medicine Agency. Indeed, the NOTE AIFA are a tool specifically introduced with the aim of constraining healthcare drug related costs and they do not represent a guideline for pharmacological therapy, although they are based on appropriateness criteria and scientific evidence. Notwithstanding, even the most recent international guidance for PPI use is in overall agreement with NOTE AIFA recommendations (NHS Barnsley Clinical Commissioning Group, 2018).

We found a high prevalence of older patients prescribed with drugs for GERD (about the 50%), confirming an evidence already reported in the literature (Franchi et al., 2020; Lassalle et al., 2020). The assessment of appropriateness, on the other hand, may vary depending on the different setting and the criteria used (Tosetti and Nanni, 2017; Franchi et al., 2020; Lassalle et al., 2020). Using the developed algorithm, we found that only around 40% of older patients prescribed with drugs for GERD in the LHUs of Bergamo and Caserta presented a proper indication for treatment according to NOTE AIFA, in particular to prevent gastrointestinal bleeding in older patients (aged 75 years or more) chronically prescribed with ASA or NSAIDs. In our opinion, this result may be suggestive of a precautionary attitude of GPs toward their patients. Indeed, according to scientific evidences, the use of anticoagulants and advanced age were found to be predisposing conditions to the risk of developing serious complications of the upper gastrointestinal tract, but these conditions should not be considered mandatory recommendation for treating all the older patients.

Even the large differences observed between Bergamo (46%) and Caserta (37%), these results pointed out how the inappropriateness of prescription is anyway high, thus the consequences are actually very similar in practice. Using administrative database, an underestimation of patients prescribed accordingly to NOTE AIFA may not be excluded, due to some limitations in assessing chronic clinical conditions. However, such a high prevalence of inappropriate prescriptions should be a huge concern not only for NHS costs but also for public health.

Voukelatou et al. found that among older patients admitted to internal medicine hospital wards, only 16% of patients (37 out of 232) receiving PPIs presented an indication in accordance to recommendations (Voukelatou et al., 2019). In a recent study in the adult French population newly prescribed PPIs, Lassalle et al. found that a general indication for use (HP eradication, co-prescription with NSAIDs, anticoagulants and/or corticosteroids or with chemotherapy or radiotherapy in patients with cancer) was present in about 70% of cases, although only a small proportion of these patients had received a procedure for investigating the digestive tract (2.3%) and near 80% among ASA and NSAIDs users did not present additional risk factors (Lassalle et al., 2020). On the contrary, Tosetti et al. found higher appropriateness of PPI use in an Italian study (near 70%). Specifically, they found a higher proportion of patients with refundable prescriptions for drugs for peptic ulcer or GERD (51.6%), probably because they used GPs medical charts to ascertain diagnosis and procedures, thus avoiding the limitations of administrative data (Tosetti and Nanni, 2017).

On the other hand, we also identified about 15% of patients potentially eligible for treatment who were not actually treated, especially in Bergamo (N = 25105, 18.1%). However, it should be considered that conditions in NOTA one indicate potential increased risk factors for gastrointestinal bleeding, but they are not mandatory recommendations for treating patients.

### Strengths and Limitations

To our knowledge, this is among the first attempts to develop an algorithm to assess drug appropriateness by integrating data from different sources of data collected for administrative purpose, thus identifying as better as possible all the potential indications for prescriptions of drugs.

The main advantage of using data collected for administrative purpose lies in the opportunity to investigate prevalence, adherence and appropriateness of medication use in the general population (Piergentili, 2006; Parabiaghi et al., 2011; Franchi et al., 2012; Bilotta et al., 2013; Franchi et al., 2013; Antoniazzi et al., 2019; Franchi et al., 2019b; Kim and Chung, 2020), in order to provide information on the context which could benefit from educational interventions. On the other hand, the use of these data presents some limitations: the lack of information on the indication to treatment and the difficulties in capturing accurately the outpatient’s clinical diagnosis. This may be even more relevant for the diagnosis of GERD and other conditions of the upper gastrointestinal tract, which are not life-threatening diseases and do not require acute hospitalizations. Furthermore, a poor reporting of such comorbidities cannot be excluded, being influenced by more severe conditions in pursuit of higher reimbursement. Based on these considerations, and differently from other experiences (Lassalle et al., 2020), we searched for hospital diagnoses and outpatient diagnostic tests up to 2 years before the first prescription with a drug for GERD. Although the results of diagnostic tests were not available, these procedures were likely prescribed by a gastroenterologist to confirm diagnosis, when supported by specific clinical symptoms. Moreover, many of these procedures (such as endoscopy) were invasive or discomforting for patients, and therefore probably prescribed only if strictly necessary. Indeed, although data on prevalence of GERD were generally scanty and showed a high uncertainty and variability—ranging from less than 20% up to 28% - due to the lack of a standardized definition and of a diagnostic gold standard for this disorder, we cannot exclude an underestimation of GERD in our population of older people (Valle et al., 1999; Zagari et al., 2008; Tosetti and Nanni, 2017; Eusebi et al., 2018; Bert et al., 2021). Also, the Global Burden of disease (GBD) included Italy among the countries with highest prevalence of GERD and stated how it increase with age showing a peak among those aged 75–79 years (GBD 2017 Gastro-oesophageal Reflux Disease Collaborators, 2020). Furthermore, the algorithm may not correctly identify all patients in chronic therapy with ASA because, due to its low cost, they might decide to buy it out of their own pocket. This is true also for many diagnostic procedures that may require long waiting times through the NHS. This is particularly evident in the Caserta LHU, where we found very low prevalence of patients with a diagnosis of GERD or related diseases both according to NOTA 1 and 48. Notwithstanding these limitations, our methodology could represent a useful, easily implementable, and low-cost instrument to limit the excessive inappropriate use of these drugs.

## Conclusion

Our results suggested a high prevalence of inappropriate use of drugs for GERD in Italy.

Much more efforts should be made to make physicians, and in particular GPs, aware of the need to contain prescriptions of drugs for GERD without proper indication and to regularly review therapy to their patients, which in turn would result in fewer potential risks for multimorbid and frail old population and in decreased costs for the NHS.

## Data Availability

The original contributions presented in the study are included in the article/Supplementary Material, further inquiries can be directed to the corresponding author.
