# Supplementary material for: Prescription Appropriateness of Drugs for Peptic Ulcer and Gastro-Esophageal Reflux Disease: Baseline Assessment in the LAPTOP-PPI Cluster Randomized Trial
Source: Front Pharmacol. 2022 Mar 28;13:803809. doi: 10.3389/fphar.2022.803809 (PMC8996306; doi:10.3389/fphar.2022.803809)
Supplement: Supplementary file 1 [file Table1.docx]

**Table S1: ICD-9-CM codes and diagnostic procedures used to develop the algorithm**

| ICD-9-CM Diseases | Outpatient services |
| --- | --- |
| 041.86 - Helicobacter pylori infection | 24H Esophageal pH monitoring |
| 456.0-2 - [Esophageal varices](https://en.wikipedia.org/wiki/Esophageal_varices) w/wo/ [bleeding](https://en.wikipedia.org/wiki/Bleeding) | Esophagogastroduodenoscopy w/wo/ biopsy |
| 530.* (excl. 3, 5) - Disease of the esophagus  (excl. Stenosis, dyskinesia, diverticulum and unspecified disorders) | Colonscopy |
| 531.* - Gastric ulcer | Endoscopic biopsy of the large intestine |
| 532.* - Duodenal ulcer | Esophagram w/ contrast/double contrast |
| 533.* - Peptic ulcer (site unspecified) | Upper and lower gastrointestinal tract radiography/series w/wo/ double contrast |
| 534.* - Gastrojejunal ulcer | Breath Test for lactose |
| 535.*1 – Gastritis and duodenitis w/ hemorrhage | Double-contrast small bowel enteroclysis |
| 569.* - [Other disorders of intestine](https://en.wikipedia.org/w/index.php?title=Other_disorders_of_intestine&action=edit&redlink=1) | Esophageal motility study |
| 578.* - [Gastrointestinal hemorrhage](https://en.wikipedia.org/wiki/Gastrointestinal_hemorrhage) | Other diagnostic procedures on the digestive system |
|  | Fecal occult blood |
| ICD-9-CM Procedures and Interventions | Fecal sample culture (Stool) test |
| 43.* Incision and Excision of Stomach | Vasoactive intestinal polypeptide |
| 44.* Other Operations on Stomach | Helicobacter Pylori antibody and culture |
|  | Evaluation of gastrointestinal bleeding |
|  | Gastrointestinal histo-cytopathology |
