# Supplementary figures and images for: Prescription Appropriateness of Drugs for Peptic Ulcer and Gastro-Esophageal Reflux Disease: Baseline Assessment in the LAPTOP-PPI Cluster Randomized Trial
Source: Front Pharmacol. 2022 Mar 28;13:803809. doi: 10.3389/fphar.2022.803809 (PMC8996306; doi:10.3389/fphar.2022.803809)

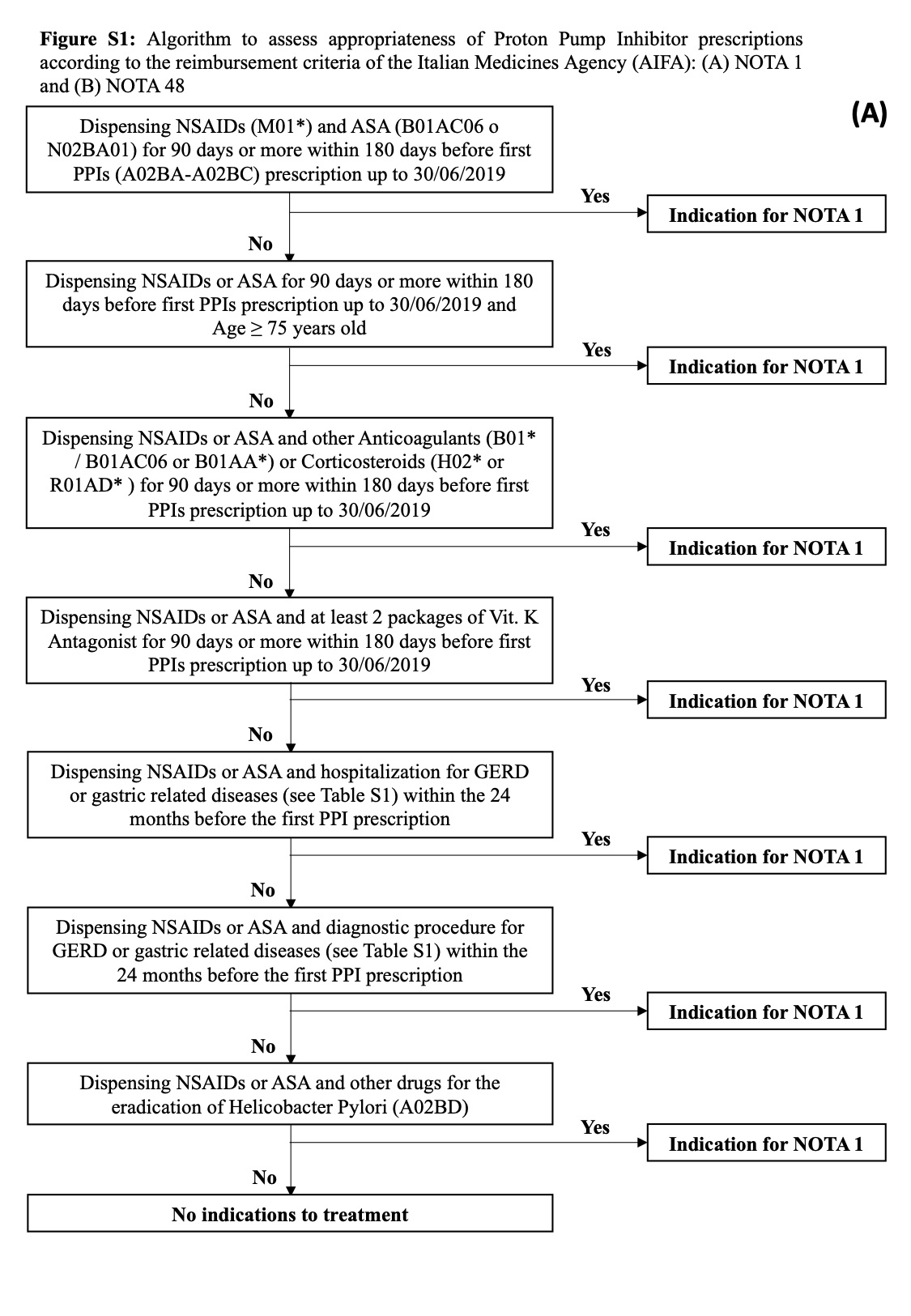

Supplement: Supplementary file 2 [file Image1.jpeg]

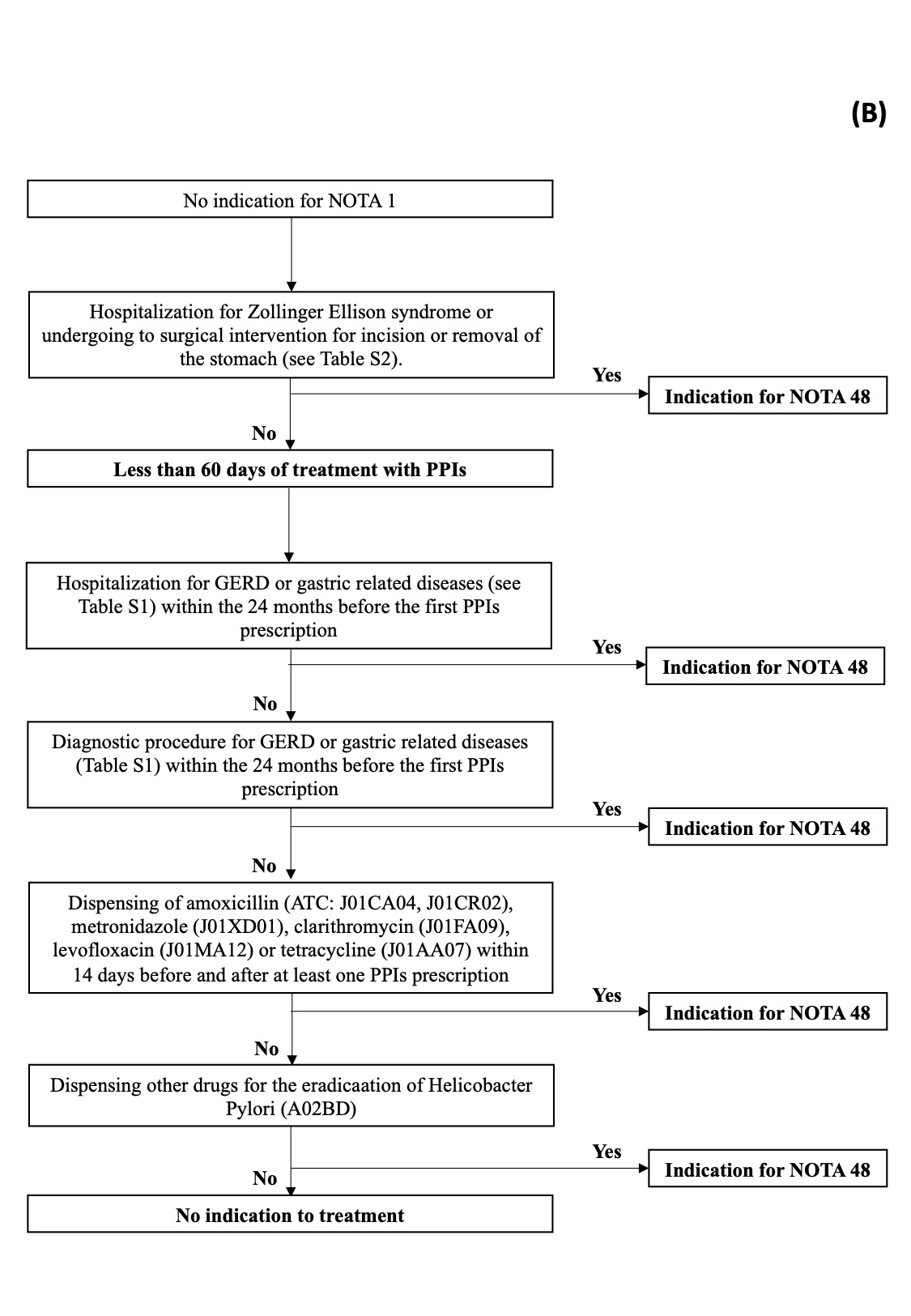

Supplement: Supplementary file 3 [file Image2.jpeg]
